# Applications and accuracy of 3D‐printed surgical guides in traumatology and orthopaedic surgery: A systematic review and meta‐analysis

**DOI:** 10.1002/jeo2.12096

**Published:** 2024-08-12

**Authors:** Silvan Hess, Julius Husarek, Martin Müller, Sophie C. Eberlein, Frank M. Klenke, Andreas Hecker

**Affiliations:** ^1^ Department of Orthopaedic Surgery and Traumatology, Inselspital, Bern University Hospital University of Bern Bern Switzerland; ^2^ Faculty of Medicine University of Bern Bern Switzerland; ^3^ Faculty of Medicine Medical University of Sofia Sofia Bulgaria; ^4^ Department of Emergency Medicine, Inselspital, Bern University Hospital University of Bern Bern Switzerland

**Keywords:** 3D printing, computer‐aided design, computer‐assisted surgery, meta‐analysis, orthopaedic procedures, orthopaedics, precision medicine, systematic review, traumatology

## Abstract

**Background:**

Patient‐Specific Surgical Guides (PSSGs) are advocated for reducing radiation exposure, operation time and enhancing precision in surgery. However, existing accuracy assessments are limited to specific surgeries, leaving uncertainties about variations in accuracy across different anatomical sites, three‐dimensional (3D) printing technologies and manufacturers (traditional vs. printed at the point of care). This study aimed to evaluate PSSGs accuracy in traumatology and orthopaedic surgery, considering anatomical regions, printing methods and manufacturers.

**Methods:**

A systematic review was conducted following the Preferred Reporting Items for Systematic Reviews and Meta‐Analysis guidelines. Studies were eligible if they (1) assessed the accuracy of PSSGs by comparing preoperative planning and postoperative results in at least two different planes (2) used either computer tomography or magnetic resonance imaging (3) covered the field of orthopaedic surgery or traumatology and (4) were available in English or German language. The ‘Quality Assessment Tool for Quantitative Studies’ was used for methodological quality assessment. Descriptive statistics, including mean, standard deviation, and ranges, are presented. A random effects meta‐analysis was performed to determine the pooled mean absolute deviation between preoperative plan and postoperative result for each anatomic region (shoulder, hip, spine, and knee).

**Results:**

Of 4212 initially eligible studies, 33 were included in the final analysis (8 for shoulder, 5 for hip, 5 for spine, 14 for knee and 1 for trauma). Pooled mean deviation (95% confidence interval) for total knee arthroplasty (TKA), total shoulder arthroplasty (TSA), total hip arthroplasty (THA) and spine surgery (pedicle screw placement during spondylodesis) were 1.82° (1.48, 2.15), 2.52° (1.9, 3.13), 3.49° (3.04, 3.93) and 2.67° (1.64, 3.69), respectively. Accuracy varied between TKA and THA and between TKA and TSA.

**Conclusion:**

Accuracy of PSSGs depends on the type of surgery but averages around 2–3° deviation from the plan. The use of PSSGs might be considered for selected complex cases.

**Level of Evidence:**

Level 3 (meta‐analysis including Level 3 studies).

**Abbreviations**
3D: three‐dimensional
aTSA: anatomic total shoulder arthroplasty
CT: computer tomography
DLP: digital light processing
FDM: fused deposition modelling
HTO: high tibia osteotomy
MRI: magnetic resonance tomography
PJ: PolyJet
PPC: printing at the point of care
PSSG: Patient‐Specific Surgical Guides
rTSA: reverse total shoulder arthroplasty
SLA: stereolithography
SLS: selective laser sintering
THA: total hip arthroplasty
TKA: total knee arthroplasty
TSA: total shoulder arthroplasty
UKA: unicompartmental knee arthroplasty

## INTRODUCTION

Three‐dimensional (3D) printing, also known as additive manufacturing or rapid prototyping, has been around for several decades and is an established manufacturing method for orthopaedic instruments and implants [6, 59]. Recently, there has been a renewed interest in 3D printing in the orthopaedic community, driven by falling costs, increasing availability of 3D printers, print materials and accessible software, as well as the goal of providing a more patient‐specific treatment. Driven by this development, so‐called ‘in house printing’ or ‘printing at the point of care’ (PPC) units have been established, where a team of physicians' designs, plans and prints surgical guides, instruments or even implants at the point of care. The aim of these units is not to complete with traditional manufacturers, but to enable a more personalized medicine and/or to address specific needs of treating physicians that cannot be met by traditional manufacturers. Regardless of the manufacturer (traditional or PPC units), the promoted benefits of 3D printed Patient‐Specific Surgical Guides (PSSGs) include reduced surgery time, minimized radiation exposure, improved intraoperative accuracy and improved patient outcomes [8, 33, 37, 49, 62]. In addition, the advantage of PPC units for orthopaedic surgeons is complete control over surgical planning, PSSGs design and manufacturing.

From an orthopaedic surgeon's point of view, the main benefit of PSSGs is high accuracy with which the preoperative plan can be transferred to the operating theatre. Moreover, these PSSGs enable complex surgeries such as deformity corrections involving more than one plane, which cannot be performed accurately by conventional techniques. However, while there are some published meta‐analyses on the accuracy of PSSGs for specific applications, the accuracy in general is not well established [5, 7, 10]. Furthermore, it is unknown whether accuracy varies between different anatomic locations, the 3D printing technology used and/or the manufacturer (traditional manufacturer vs. PPC unit). However, this information seems important for surgeons and hospitals considering setting up a PPC unit or using PSSGs from a traditional manufacturer. It may also be relevant to research groups developing new treatments based on 3D printing. The aim of this review therefore was to

I) collect data on the used printing technology and the type of surgery performed with the aid of PSSGs,
II) assess the accuracy of PSSGs in traumatology and orthopaedic surgery,
III) assess, if there are differences in accuracy if PSSGs are used in different locations and/or produced by traditional manufacturers or PPC units.

## METHODS

### Protocol and registration

The electronic databases PROSPERO and Cochrane were searched for prior registered, on‐going or published study protocols, and systematic reviews according to this topic but no study was found. To conduct this systematic review, the Cochrane Collaboration Handbook was consulted and reported in accordance with the Preferred Reporting Items for Systematic Reviews and Meta‐Analysis (PRISMA) guidelines [24, 41].

### Inclusion and exclusion criteria

Studies were eligible for inclusion if meeting the following inclusion criteria: (1) the study assessed the accuracy of 3D‐printed surgical guides by comparing preoperative planning and postoperative results in at least two planes, (2) computer tomography or MRI was used for the assessment, (3) performed in the field of Orthopaedic surgery and/or traumatology, (4) were available in English or German. Studies were excluded despite meeting these inclusion criteria if one of the following exclusion criteria were met: in vitro studies (e.g., animal studies, cadaver studies), studies including less than 15 participants, studies including patients under the age of 18, reviews, case reports, letters to the editor, congress abstracts, commentaries and patents.

### Information sources

The electronic databases OvidSP Embase, OvidSP MEDLINE, Cochrane Library and Scopus were searched from database inception to the search date (23 November 2022 and updated 18 October 2023) for studies meeting the above‐mentioned inclusion criteria.

### Search strategy

The search strategy was designed by Julius Husarek via a text analysis of key studies. Medical subject headings (e.g., Emtree and MeSH), proximity operators (NEAR, NEXT, ADJ and W/), truncation/wildcard symbols and Boolean operators (OR, AND and NOT) were used. The following keywords were used: accuracy; 3D printing; surgical guide; traumatology; orthopaedic surgery. After conducting the preliminary search in Embase via the Ovid platform, the strategy for the other databases was adapted. The final search strategy can be found in Supporting Information S1: Appendix 1.

### Selection process

Results of the different databases were collected and checked for duplications using the method described by Bramer et al. within EndNote 20.5 [3]. The remaining articles were entered into the web‐based platform Rayyan to accelerate and assist the screening process with collaboration among the authors [40].

The titles and abstracts of each article were screened by two independent reviewers (Julius Husarek and Silvan Hess). Disagreement at the title and abstract stages were resolved by automatic inclusion to ensure thoroughness. Disagreement at the full text stage were resolved by consensus between the two reviewers. If a consensus could not be reached, a third, more senior reviewer (Andreas Hecker) helped to resolve the discrepancy. The references of included articles were then screened to capture any articles that may have been missed and one additional article was found.

### Data collection process

Data were extracted and recorded into a predefined data collection form in Microsoft Excel (Microsoft Office Packages) by two reviewers (Julius Husarek and Silvan Hess). For each type of surgery, specific values were defined to judge the accuracy of PSSG. Table 1 shows a detailed list of these values for each surgery. The used printing technology was categorized according to Table 2.

**Table 1:** List of parameters for each type of surgery.

Surgery	Required parameters
THA cup	Angular deviation:
	Inclination (coronal plane)
	Version (axial plane)
	Displacement: Centre of rotation
THA stem	Angular deviation:
	Version (axial plane)
	Femoral neck angle
TKA femoral component	Angular deviation:
	Coronal (lateral distal femoral angle)
	Sagittal (flexion/extension angle)
	Axial (posterior condylar twist angle)
TKA Tibia component	Angular deviation:
	Coronal (medial proximal tibial angle)
	Sagittal (posterior slope)
	Axial
Dorsal spondylosis	Angular deviation:
	Sagittal
	Axial
	Displacement: Entry point or Narrowest point of pedicle
TSA Glenoid component	Angular deviation:
	Inclination (coronal plane)
	Version (axial plane)
	Displacement: Position of the glenoid component

Abbreviations: THA, total hip arthroplasty; TKA, total knee arthroplasty; TSA, total shoulder arthroplasty.

**Table 2:** List of commonly used 3D printing technology, brief description of each technique and examples.

Printing technology	Description	Example of printer (company)	Example of printing material (company)
Fused deposition modelling (FDM)	A printing head extrudes a thermoplastic filament, which is melted during the extrusion processes and becomes solid when applied.	AON‐M2 2020 (AON3D)	KetaSpire CF10 LS1 (Solvay)
Stereolithography (SLA)	A laser is used to cure liquid resin into solid objects. The solid part of the object is secured to a building platform, which is elevated/pulled out of a basin of resin.	Form3B (Formlabs)	BioMed Amber Resin (Formlabs)
Selective laser sintering (SLS)	A laser is used to cure powdered material into solid objects. A tank is filled with powder is hardened by a laser.	Formiga P110 (EOS)	PA 2200 (EOS)
Digital light processing (DLP)	Similar to the SLS technique but a projector is used instead of a laser to flash an image of each layer of the object onto the surface of the resin.	Primeprint (Dentsply Sirona)	Primeprint guide resin (Dentsply Sirona)
PolyJet (PJ)	Tiny droplets of liquid photopolymer are dropped onto a build tray and then cured with UV light.	Stratasys J5 MediJet™ 3D printer (Stratasys Ltd)	MED 610™ (Stratasys Ltd)

### Quality assessment

The ‘Quality Assessment Tool for Quantitative Studies’ developed by the Effective Public Health Practice Project Canada was used to assess the methodological quality of the studies as recommended by the Cochrane Collaboration [53].

### Data synthesis

The statistical analysis was performed with Stata, version 16.1 (StataCorp LLC). Random effects meta‐analyses to determine the pooled mean absolute deviation with 95% confidence interval (CI) from the 3D plan in degree were performed for each studied anatomic region (shoulder, hip, spine and knee). The estimates were stratified according to type of surgery. Stata's test of group differences was performed to evaluate differences between the type of surgeries within one region. Additionally, for knee arthroplasty a meta‐analyses was performed with the mean deviation (including negative values) as an estimate. If the overall test of group differences was significant and more than two subgroups were present, pairwise comparisons were made.

Last, three region comparisons (i) shoulder versus hip; (ii) shoulder versus knee and (iii) hip versus knee using a random effects model stratified by the regions. The subgroup means were compared with a test of group differences and the difference of the pooled means was calculated.

If a study appeared multiple times in a subgroup, for example, multiple accuracy measures were presented in this study, all presented surrogates for accuracy in a study were pooled using a fixed effects model to obtain one aggregated (aggr.) estimate per study in a subgroup.

A *p*‐value < 0.05 was considered significant. No *p*‐value adjustment for multiple testing was performed. Heterogeneity among studies was assessed with *I* ^2^ statistics.

## RESULTS

### Search results

**Figure 1:**
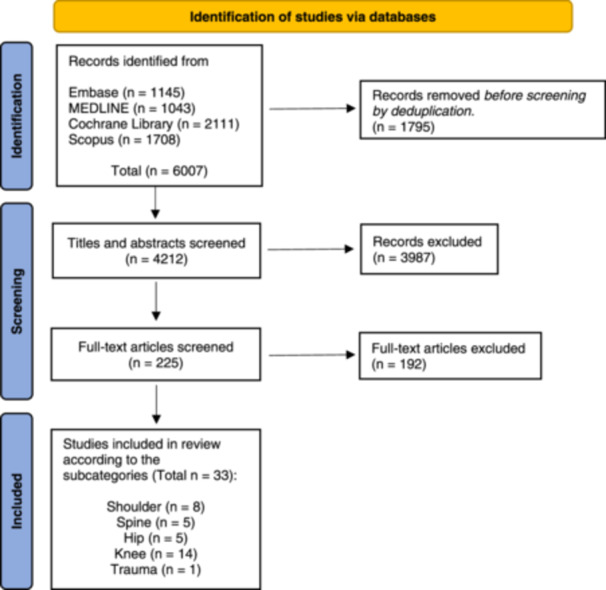
Preferred Reporting Items for Systematic Reviews and Meta‐Analysis (PRISMA) flow diagram.

The search yielded 6007 publications. Using the method described above, 1795 duplicates were identified, resulting in 4212 studies eligible for screening. A total of 3987 papers were excluded after title and abstract screening. The full‐text of the remaining 225 articles was assessed and 33 articles were included. The PRISMA flowchart is shown in Figure 1. Table 3 shows all demographic and technical information.

**Table 3:** Demographic and technical information.

Reference	Country	# P	# Male	# Female	Category	Imaging modality
Pijpker et al. [44]	Netherlands	15	5	10	Spine	CT
Gauci et al. [20]	France	17	3	14	Shoulder	CT
Wang et al. [58]	China	17	5	22	THA	CT
Fujita et al. [19]	Japan	17	14	3	Spine	CT
Dallalana et al. [13]	Australia	20	N/A		Shoulder	CT
Yamamura et al. [61]	Japan	21	N/A		TKA	CT
Vervaecke et al. [56]	Belgium	22	17	5	Shoulder	CT
Fucentese et al. [18]	Switzerland	23	16	7	HTO	CT
Hananouchi et al. [22]	Japan	24	2	22	THA	CT
Kerens et al. [30]	Netherlands	25	15	11	UKA	MRI, CT
Boonen et al. [1]	Netherlands	26	13	13	TKA	MRI, CT
Yamamura et al. [60]	Japan	30	9	21	TKA	CT
Verborgt et al. [55]	Belgium	33	9	24	Shoulder	CT
Marcoin et al. [36]	France	35	25	10	Shoulder	CT
Ferretti et al. [17]	Italy	36	19	17	THA	CT
Matsukawa et al. [37]	Japan	43	24	19	Spine	CT
Chaouche et al. [9]	France	71	45	26	HTO	CT
Spencer‐Gardner et al. [49]	Australia	100	55	45	THA	CT
Jacquet et al. [28]	France	100	59	41	HTO	CT
Crane et al. [11]	Australia	217	N/A		TKA	MRI, CT
Hendel et al. [23]	USA	31 (15 PSI)	N/A		Shoulder	CT
Small et al. [48]	USA	36 (18 PSI)	20 (10 PSI)	16 (8 PSI)	THA	CT
Kwak et al. [33]	Korea	39 (19 PSI)	9 (4 PSI)	30 (15 PSI)	Shoulder	CT
Parratte et al. [42]	France	40 (20 PSI)	N/A		TKA	MRI, CT
De Vloo et al. [15]	Belgium	50 (25 PSI)	21 (11 PSI)	29 (14 PSI)	TKA	MRI, CT
Sun et al. [51]	China	54 (20 PSI)	31 (11 PSI)	23 (9 PSI)	Traumatology	CT
Ng et al. [39]	USA	78 (51 PSI)	26	52	TKA	MRI, CT
Sariali et al. [47]	France	80 (40 PSI)	23 (7 PSI)	57 (33 PSI)	TKA	CT
Cui et al. [12]	China	84 (42 PSI)	39 (20 PSI)	45 (22 PSI)	Spine	CT
Roh et al. [45]	Korea	90 (42 PSI)	8 (3 PSI)	82 (39 PSI)	TKA	CT
Dasari et al. [14]	USA	36 (19 PSI)	25 (13)	11 (6)	Shoulder	CT
Mohar et al. [38]	Slovenia	39	11	28	Spine	CT
Van Genechten et al. [54]	Belgium	30	27	3	HTO	CT

Abbreviations: CT, computer tomography; HTO, high tibia osteotomy; MRI, magnetic resonance tomography; PSI, patient‐specific instrumentation; THA, total hip arthroplasty; TKA, total knee arthroplasty; TSA, total shoulder arthroplasty.

### Quality assessment

The methodological quality was considered ‘strong’ in 11 studies [9, 11, 12, 14, 18, 19, 23, 33, 43, 47, 51], ‘moderate’ in 19 studies [1, 13, 15, 20, 28, 30, 37, 38, 39, 44, 45, 48, 49, 54, 55, 56, 58, 60, 61], and ‘weak’ in three studies [17, 22, 36]. Detailed results from the quality assessment can be found in Supporting Information S2: Appendix 2.

### Printer and resin

In 12 studies, the surgical guides were designed and printed at the point of care [12, 13, 22, 23, 33, 44, 48, 51, 58]. The used printer was reported in eight out of these 12 studies [14, 22, 23, 33, 38, 48, 51, 54]. PJ (3), SLS (3) and SLA (2) printing technologies were used. Printer name and brand were reported by six studies [14, 23, 33, 38, 48, 51]. In the remaining 21 studies, the surgical guides were designed in collaboration with a company and manufactured by the company. The used technology was reported by three studies (all used SLS printer). The exact name of the used printer and resin was reported by two studies [18, 20]. The remaining companies were thus contacted to collect data on the used printer and resin. None of the companies provided detailed information but two agreed to share the type of printer (SLS) and the material (Polyamid). Additionally, Zimmer Biomet and Corin declared a partnership with Materialise, whereby Materialise is responsible for the manufacturing process. Upon request, Materialise did not report any details regarding the process either. Various programmes were used for the 3D segmentation, planning and assessment but the software provided by Materialise was the most used. A detailed list of the used printer, resign and software is shown in Tables 4 and 5.

**Table 4:** Software used for preoperative planning and postoperative evaluation by the studies.

Reference	Pre‐op planning software (company)	Post‐op evaluation software (company)
Boonen et al. [1]	Mimics and 3‐matic (Materialise)	Mimics and 3‐matic (Materialise)
Chaouche et al. [9]	NA	NA
Crane et al. [11]	Specific Instrumentation (Zimmer Biomet)/Visionaire PSI (Smith and Nephew)	NA
Cui et al. [12]	Mimics (Materialise), NA (Geomagic software)	NA
Dallalana et al. [13]	SurgiCase Connect software (Materialise)	NA
De Vloo et al. [15]	Signature Personalized Patient Care System (Biomet Inc)*/Mimics Innovation Suite (Materialise)	Mimics (Materialise)
Ferretti et al. [17]	Optimized Positioning System (OPS, Corin Ltd.)	NA
Fucentese et al. [18]	CASPA (Balgrist CARD AG)	mediCAA module osteotomy (Hectec GmbH) and CASPA (Balgrist CARD AG)
Fujita et al. [19]	MySpine (Medacta International AG)	Solidworks Software (Dassault Systemes Company)
Gauci et al. [20]	Glenosys (Imascap)	Amira (Visualization Sciences Group)
Hananouchi et al. [22]	Virtual Place‐M (Medical Imaging Laboratory), Visualization Toolkit libraries (Kitware), Magics 11 (Materialise)	NA
Hendel et al. [23]	ArthroPlan (Arthromeda Inc)	ArthroPlan (Arthromeda Inc)
Jacquet et al. [28]	NA	NA
Kerens et al. [30]	Materialise NV (Materialise NV)	NA
Kwak et al. [33]	Mimics (Materialise), 3D sensor (Comet5; Carl Zeiss)	A VIEWER (Coreline Soft)
Marcoin et al. [36]	Zimmer D‐Shoulder Planning Software (Zimmer Biomet)	NA
Matsukawa et al. [37]	Mimics (Materialise), Solidworks (Dassault Sysètmes)	NA
Ng et al. [39]	Mimics and 3‐matic (Materialise)	Mimics and 3‐matic (Materialise)
Parratte et al. [42]	Mimics (Materialise)	NA
Pijpker et al. [44]	Mimics and 3‐matic (Materialise)	Mimics and 3‐matic (Materialise)
Roh et al. [45]	3D surgical planning (Materialise)	OnDemand3DTM (Cybermed Inc)
Sariali et al. [47]	Knee‐Plan and BoneSurfacer (Symbios Orthopédie SA)	Knee‐Plan (Symbios)
Small et al. [48]	Arthroplan (Custom Orthopaedic Solutions)	NA
Spencer‐Gardner et al. [49]	Optimized Positioning System (OPS, Corin Ltd.), Solidworks (Dassault Systems)	CAD v5.1 (Simpleware)/ScanIP v5.1 (Simpleware)
Sun et al. [51]	Mimics (Materialise)	NA
Verborgt et al. [55]	PSI Shoulder Segmentation Application and PSI Planner (Zimmer Biomet)	Mimics (Materialise), Na (Medical Metrics)
Vervaecke et al. [56]	PSI Shoulder Segmentation Application and PSI Planner (Zimmer Biomet)	Mimics (Materialise NV).
Wang et al. [58]	Mimics (Materialise)	NA
Yamamura et al. [60]	MyKnee (Medacta International AG)	Mimics (Materialise), NA (Geomagic software)
Yamamura et al. [61]	ZedView and ZedKnee (LEXI Co. Ltd.)	ZedView and ZedKnee (LEXI Co.)
Dasari et al. [14]	Mimics (Materialise), Rhinoceros (Robert McNeel & Associates), Tornier Blueprint (Stryker), Virtual Implant Positioning™ System Arthrex (Arthrex), DJO MatchPoint	NA
Mohar et al. [38]	3D vertebral model reconstruction (EBS 3.1.0, EKLIPTIK)	NA
Van Genechten et al. [54]	Mimics and 3‐matic (Materialise)	Mimics and 3‐matic (Materialise)

**Table 5:** Patient‐Specific Surgical Guides (PSSGs) manufacturers, printer and printing material by the studies.

Reference	PSSGs product (manufacturer)	Printer, company	Printer technology	Material, material category (manufacturer)	Implant (manufacturer)
Boonen et al. [1]	Signature Personalized Patient Care System (Biomet Inc)	NA	NA	NA	Vanguard Knee (Zimmer Biomet)
Chaouche et al. [9]	ONE—patient‐specific solution, (Newclip Technics)	NA	NA	NA	Activmotion HTO plate (Newclip Technics)
Crane et al. [11]	Zimmer PSI Knee System (Zimmer Biomet)/Visionaire PSI (Smith and Nephew)	NA	NA	NA	NexGen LPS Flex/Legion High Flex (Zimmer Biomet/Smith & Nephew)
Cui et al. [12]	PPC	NA	NA	NA	NA
Dallalana et al. [13]	PPC	NA	NA	NA	DJO Surgical Reverse Shoulder Prosthesis & DJO Surgical Turon Modular Shoulder System (DJO)
De Vloo et al. [15]	Signature Personalized Patient Care System (Biomet Inc)	NA	NA	NA	Vanguard Knee (Zimmer Biomet)
Ferretti et al. [17]	OPS PSI (Corin Ltd.)	NA	NA	NA	Trinity cup/TriFit (Corin Ltd.)
Fucentese et al. [18]	MyOsteotomy (Medacta International AG)	Formiga P110 (EOS GmbH)	SLS	PA2200, Polyamid (EOS GmbH)	Tomofix Medial High Tibial Plate (DePuy Synthes)
Fujita et al. [19]	MySpine (Medacta International AG)	NA	SLS*	PA2200, Polyamid (NA)	NA
Gauci et al. [20]	NA (Tornier SAS)	EOSINT P380 (EOS GmbH)	SLS	PA2200, Polyamid (EOS GmbH)	NA (Tornier SAS, Montbonnot)
Hananouchi et al. [22]	PPC	Eden 250 (Objet Geometries Ltd)	PJ	NA	NA
Hendel et al. [23]	PPC	NA (Astro Manufacturing & Design)	SLA	NA	Anchor‐Peg‐Glenoid (DePuy)
Jacquet et al. [28]	ONE—patient‐specific solution (Newclip Technics)	NA	NA	NA	Activmotion HTO plate (Newclip Technics)
Kerens et al. [30]	Signature Personalized Patient Care System (Biomet Inc)				Oxford UKA (Zimmer Biomet Ltd)
Kwak et al. [33]	PPC	Project 3510 (3D Systems)	PJ	VisiJet M3 Crysta, Photopolymer (3D Systems)	Equinoxe Reverse System (Exactech)
Marcoin et al. [36]	NA (Zimmer Biomet)	NA	NA	NA	Trabecular Metal Reverse Shoulder System (Zimmer Biomet)
Matsukawa et al. [37]	MySpine MC (Medacta International AG)	NA	SLS*	PA2200, Polyamid (NA)	MUST (Medacta International AG)
Ng et al. [39]	Zimmer PSI Knee System (Zimmer Biomet)	NA	NA	NA	Legac PS Flex Fix Bearing TKA with NexGen Procoat Stemm (Zimmer Biomet)
Parratte et al. [42]	Zimmer PSI Knee System (Zimmer Biomet)	NA	NA	NA	NexGen LPS Flex (Zimmer Biomet)
Pijpker et al. [44]	PPC	NA	NA	NA	NA
Roh et al. [45]	Signature Personalized Patient Care System (Biomet Inc)	NA	NA	NA	Vanguard Knee (Zimmer Biomet)
Sariali et al. [47]	Knee‐Plan (Symbios Orthopédie SA)	NA	NA	NA	FIRST (Symbios Orthopédie SA)
Small et al. [48]	PPC	SLA 5000 (3D Systems Inc)	SLA	Watershed XC11122, NA, Astro (Manufacturing)	Trident acetabular component (Stryker)
Spencer‐Gardner et al. [49]	OPS PSI (Corin Ltd)	NA	SLS	PA2200, Polyamid (NA)	NA
Sun et al. [51]	PPC	Projet HD3500 Plus (3D Systems)	PJ	VisiJet M3 Crysta, Photopolymer (3D Systems)	NA
Verborgt et al. [55]	NA (Zimmer Biomet)	NA	NA	NA	NA
Vervaecke et al. [56]	NA (Zimmer Biomet, Warsaw)	NA	NA	NA	Bigliani/Flatow TSA (Zimmer Biomet)
Wang et al. [58]	PPC	NA	SLS	NA Polyamide (NA)	NA
Yamamura et al. [60]	MyKnee (Medacta International AG)	NA	SLS*	PA2200, Polyamid (NA)	GMK Sphere (Medacta International AG)
Yamamura et al. [61]	Prophecy (MicroPort Orthopaedics)	NA	NA	NA	Evolution PS (MicroPort Orthopaedics)
Dasari et al. [14]	PPC	Formlabs Form 2 (Formlabs)	SLA	Formlabs Biomed Amber Resin (Formlabs)	
Mohar et al. [38]	PPC	EOSINT P396 (EOS)	SLS	Biocompatible polyamidePA 2200 (EOS)	CD HORIZON LEGACY or SOLER Spinal System (Medtronic)
Van Genechten et al. [54]	PPC	Unknow (OCEANZ®)		Polyamide 12 (OCEANZ®)	

*Note*: Values marked with a * were reported after contacting the corresponding company.

Abbreviations: NA, not available; PJ, PolyJet or Material Jetting; PPC, printed at the point of care unit; PSSGs, Patient Specific Surgical Guides; SLA, stereolithography; SLS, selective laser sintering.

### Shoulder surgery

**Figure 2:**
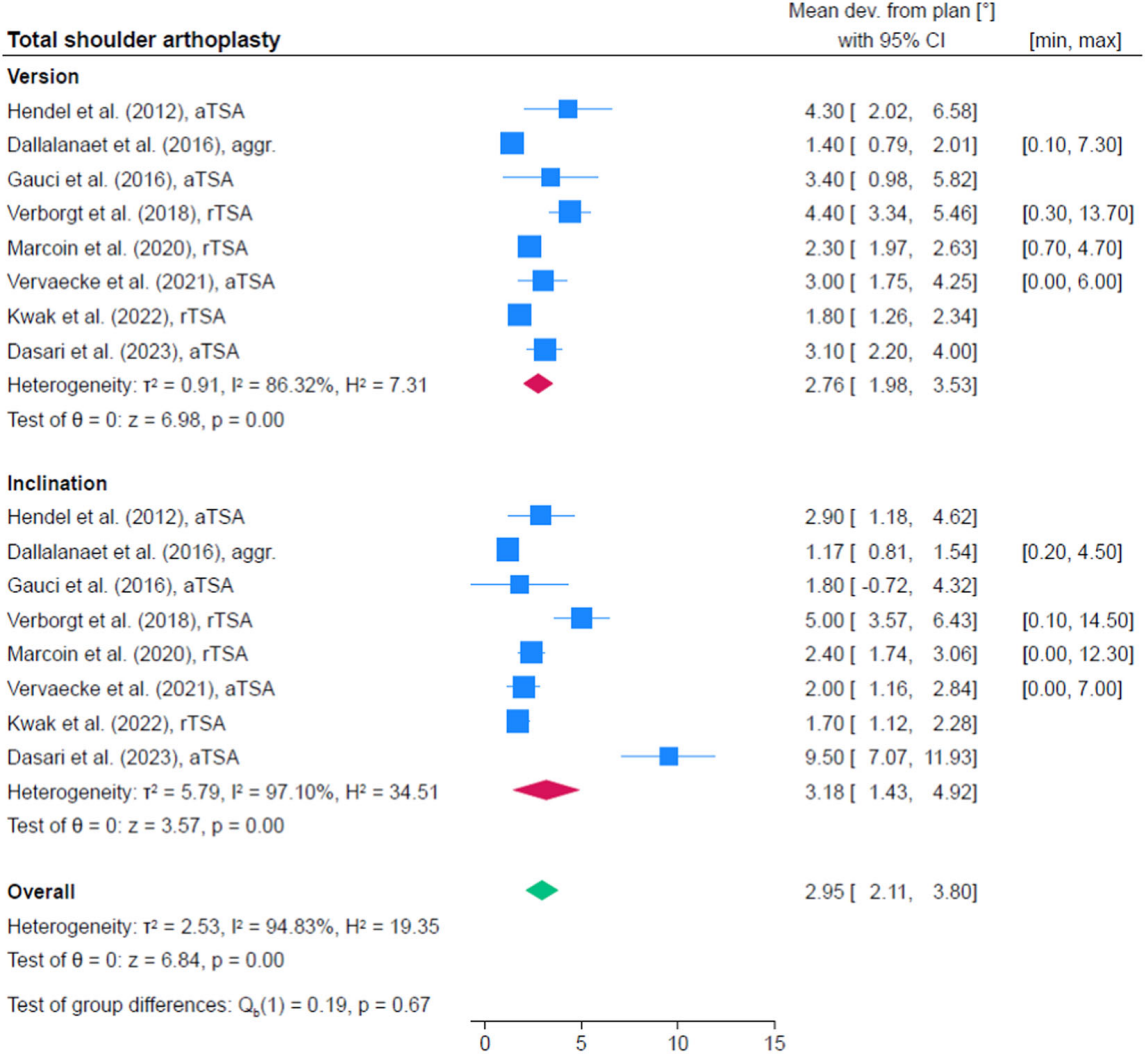
Quantitative analysis of included studies assessing the accuracy in total shoulder arthroplasty. CI, confidence interval.

Eight studies were identified within the category of shoulder surgery [13, 14, 20, 23, 33, 36, 55, 56]. Four studies focused on the investigation of anatomical total shoulder arthroplasty (aTSA) [14, 20, 23, 56], while another three studies focused on the reverse total shoulder prosthesis (rTSA) [33, 36, 55]. A single study investigated both types of prostheses [13]. All studies only assessed the glenoid component. Angular deviation (e.g., inclination and version) from preoperative plan was reported by all studies. In addition, the displacement (anterior‐posterior [AP], lateral‐medial as well as superior‐inferior [SI]) was measured in two or three different planes to determine the displacement of the glenoid component orientation in three studies [13, 20, 23]. Additionally, the deviation in screw orientation in the AP and SI direction was investigated in two different studies. Here, Kwak et al. reported that the deviation of coracoid, spinal and inferior screws in AP direction was 2.5° with a standard deviation (SD) 1.7, 3.5° (SD 2.7) and 2.9° (SD 1.8) and for SI direction of 2.4° (SD 1.9), 2.9° (SD 1.8) and 3.8° (SD 2.5) [33]. Verborgt et al. reported a superior and inferior screw with a deviation in AP direction of 2.8° (SD 2.6) and 4.1° (SD 3.1), respectively, and in SI direction of 2.8° (SD 2.6) and 5.3° (SD 2.8) [55]. Figure 2 shows the pooled accuracy of the studies regarding version, inclination and overall. The comparison in accuracy between aTSA and rTSA was not significant (*p* = 0.64 for inclination, *p* = 0.32 for version).

### Spine surgery

**Figure 3:**
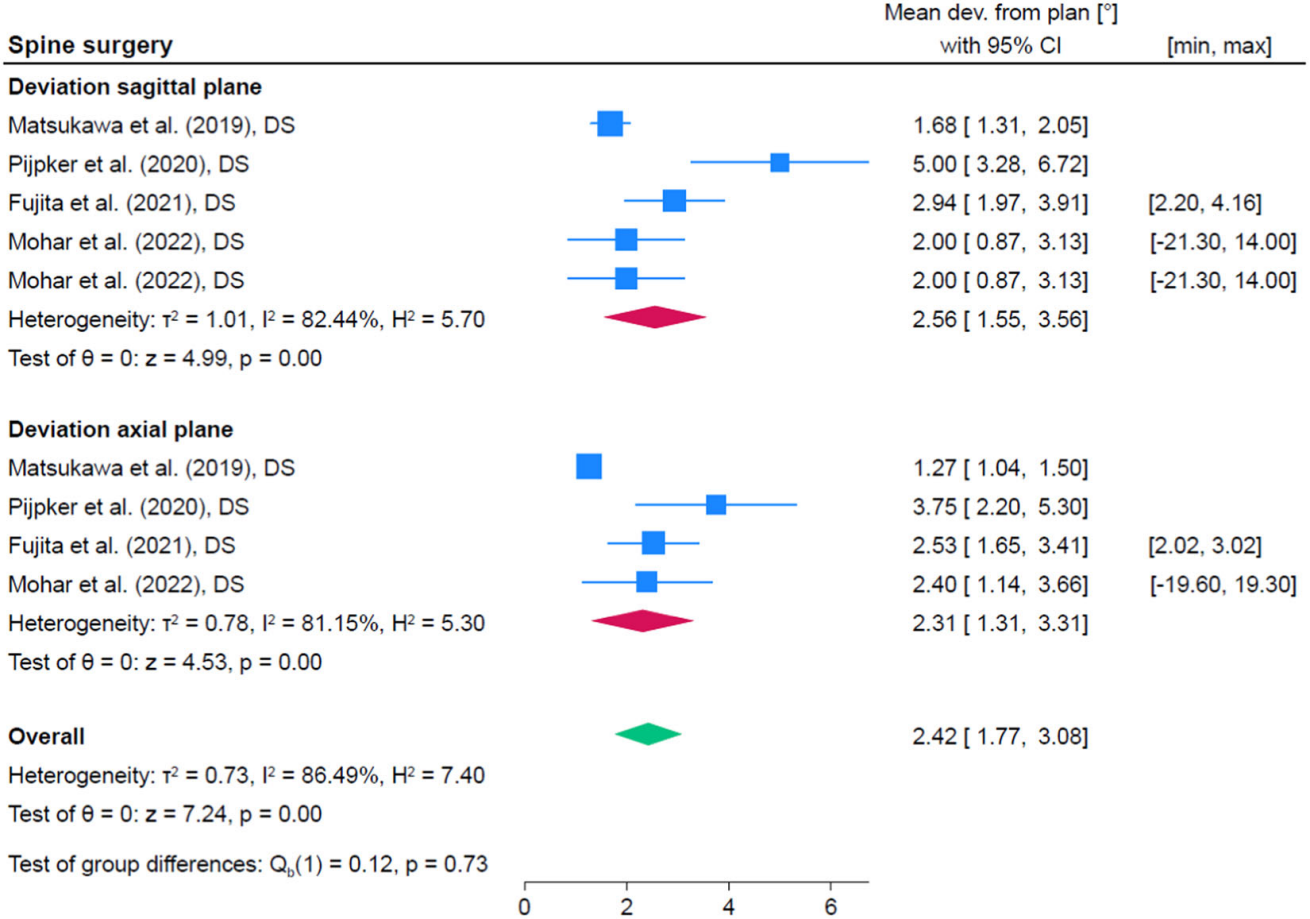
Quantitative analysis of included studies assessing the accuracy of the screw placement during spondylodesis. CI, confidence interval.

Five studies were identified within the category of spine surgery [12, 19, 37, 38, 44]. Studies assessed accuracy of pedicle screw placement in the lumbar, thoracic, cervical, and cervico‐thoracic spine. Angular deviation (deviation in sagittal and transverse plane) and displacement (SI and medial‐lateral) were reported by all studies. Figure 3 shows the pooled accuracy of the studies regarding sagittal and axial angular deviation.

Matsukawa et al. further determined the midpoint deviation from the central portion of the pedicle, which measured 0.62 mm (SD 0.42) [37]. Pijpker et al. reported an average screw entry point deviation of 1.40 mm (SD 0.81) from the preoperative planning [44]. Fujita et al. reported an average vertical deviation of the screw's entry point of 0.63 mm (SD 0.50) and horizontal deviation of 0.43 mm (SD 0.35) [19]. They further reported a vertical deviation of 0.43 mm (SD 0.30) and horizontal deviation of 0.56 mm (SD 0.43) of the screws at the narrowest point of the pedicle. Mohar et al. also reported an entry point deviation of 0.8 (SD 1.4 mm; range: −5.8 to 7.7 mm) and 0.8 (SD 1.4 mm; range: −6.1 to 6.3 mm) on the horizontal and vertical axes, respectively [38]. Cui et al. reported values for angular deviation and displacement which were 10 times smaller than the values from the remaining three studies not only for the PSSGs (sagittal plane: 0.04° ± 0.02, axial plane 0.06° ± 0.02) but also for the conventional technique (standard instrumentation and free hand: sagittal plane 0.19° ± 0.05, axial plane 0.13° ± 0.04) and was thus excluded from the data synthesis [12].

### Hip surgery

**Figure 4:**
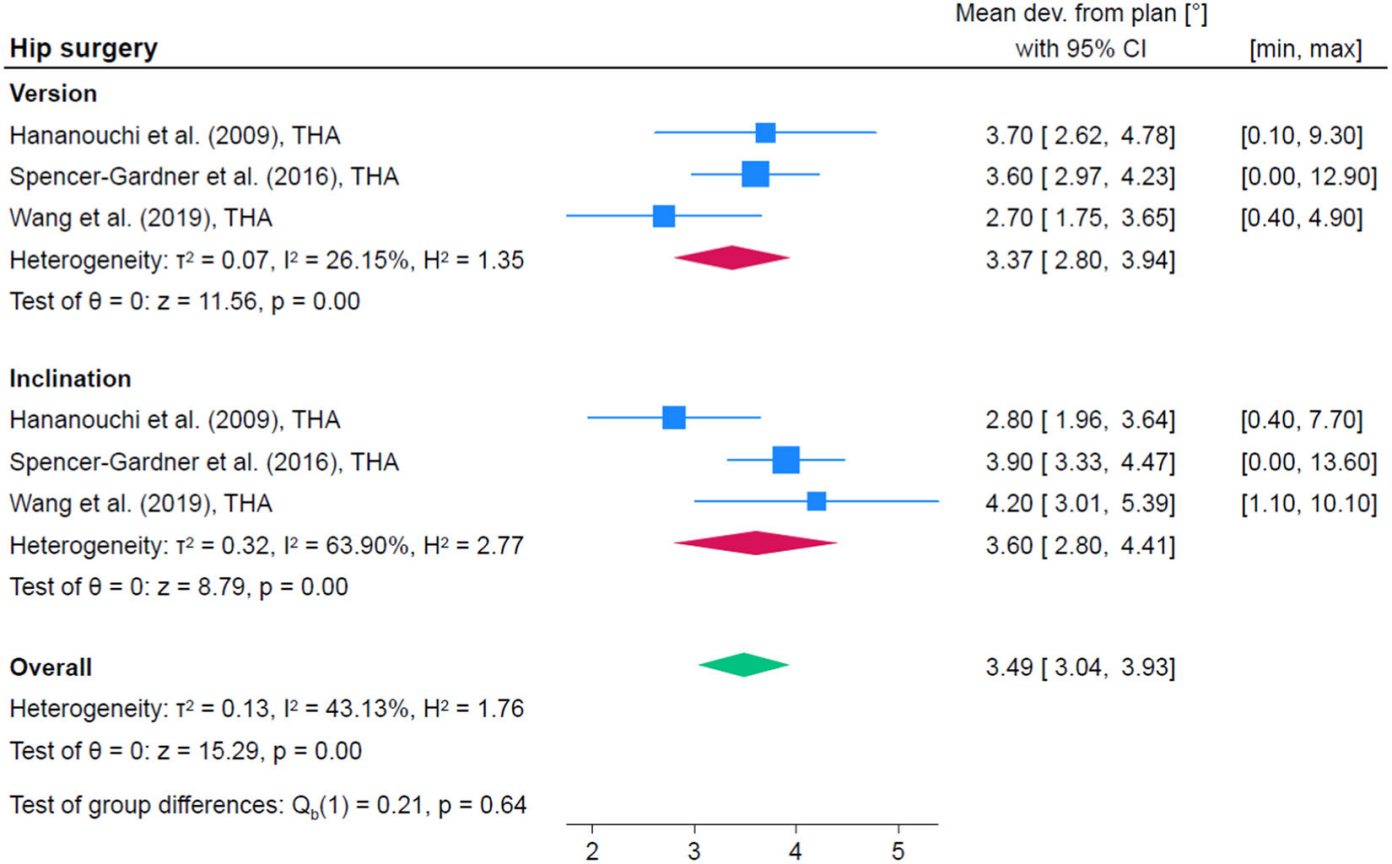
Quantitative analysis of included studies assessing the accuracy in total hip arthroplasty. CI, confidence interval.

Five studies were selected within the category hip surgery [17, 22, 48, 49, 58]. Four reported data on the accuracy of the cup placement. The study by Small et al. was excluded from the quantitative analysis because they reported negative mean differences between planned and postoperative results, which does not represent a measurement of accuracy [48]. However, their values are nevertheless interesting in the clinical setting. They reported a mean difference between planned and postoperative inclination of −2.0° (SD 7.3) and version of −0.2° (SD 6.9). Ferretti et al. additionally described the femoral osteotomy height with a deviation of 1.6 mm (range 0–4) from the preoperative planning [17]. Wang et al. reported the bilateral rotator centre discrepancy (BRCD) with a deviation of 3.38 mm (SD 3.0; range 0.9–11) [58]. Figure 4 shows the pooled accuracy of the studies regarding version, inclination and overall.

### Knee surgery

**Figure 5:**
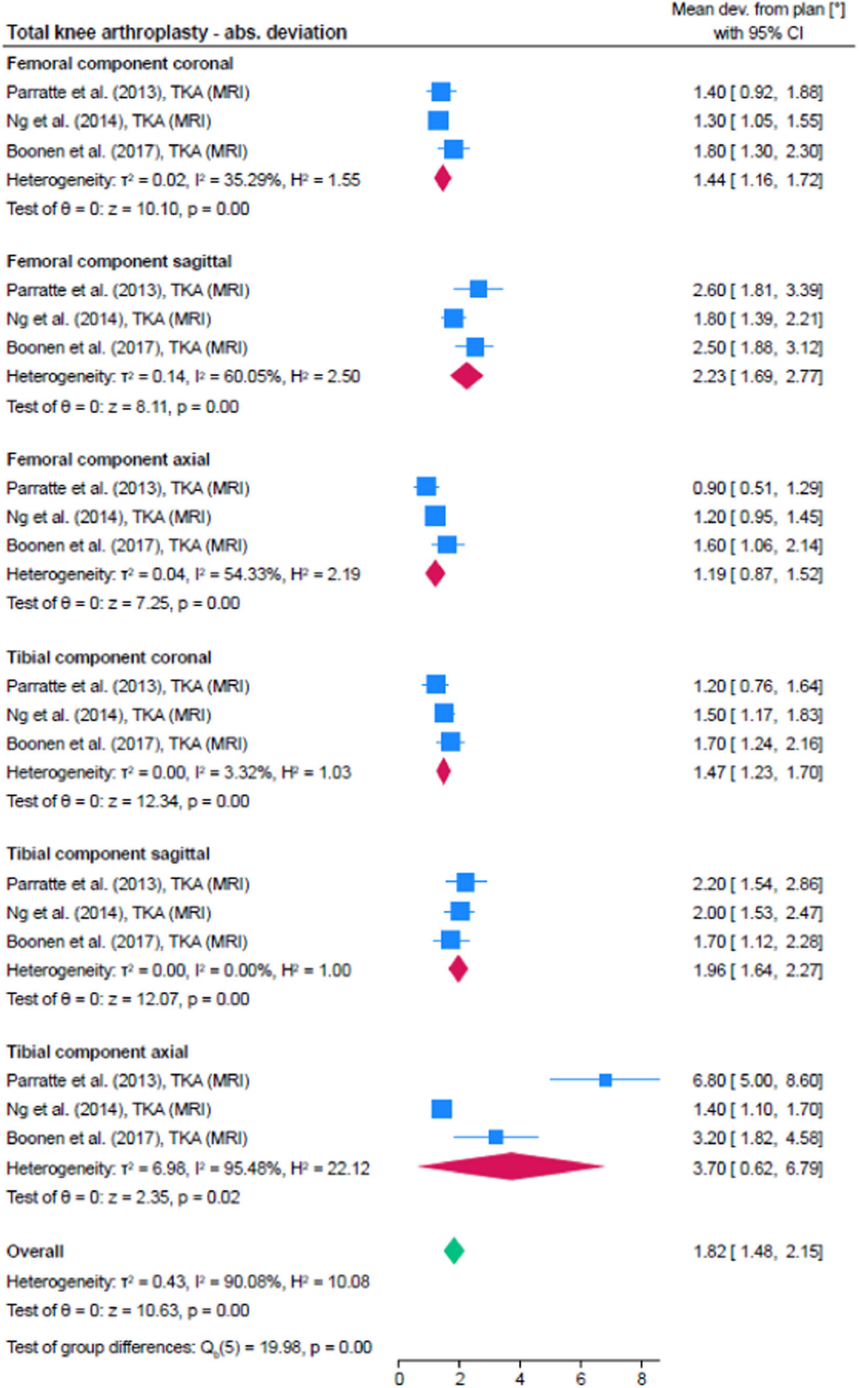
Quantitative analysis of included studies assessing the accuracy in total knee arthroplasty. CI, confidence interval; MRI, magnetic resonance tomography; TKA, total knee arthroplasty.

In total, 14 studies were identified within the category of knee surgery, whereby five assessed the accuracy of total knee arthroplasty (TKA) with the aid of surgical guides and three the accuracy of surgical guides for high tibia osteotomy and one the use of PSSGs in unicompartmental knee arthroplasty (UKA). Figure 5 shows the pooled accuracy of the studies assessing the accuracy of PSSGs in TKA for each parameter as well as overall. There were four additional studies, which assessed the accuracy of PSSGs in TKA but they reported negative mean deviations (between planned and resulting orientation of the components). This seems odd since mathematically, a measurement of accuracy cannot be less than 0 (i.e., no deviation) and the studies were thus excluded from the quantitative analysis. However, their values are nevertheless interesting and are thus shown in Table 6a,b.

**Table 6:** Studies assessing the difference between planned alignment and postoperative alignment of total knee arthroplasty (TKA).

TKA													
		Coronal		Range		Sagittal		Range		Axial		Range	
Author	Year	Mean	SD	Min	Max	Mean	SD	Min	Max	Mean	SD	Min	Max
**(a) Differences between planned and resulting position of the femur component**													
Sariali	2019	0.1	0.1	−0.3	0.5	−0.8	3.1	−1.8	0.1	0.2	1.8	−0.3	5.0
Roh	2013	1.0	1.4	−5.0	5.7	0.2	2.3	−3.2	8.0	0.5	1.8	−2.4	4.5
Crane	2014	−0.4	1.3	−5.0	3.0	0.9	1.4	−3.0	5.0	−0.2	2.0	−4.0	5.0
		−0.4	1.8	−4.0	3.0	1.3	1.9	−4.0	9.0	−0.3	2.2	−5.0	6.0
De Vloo	2017	0.1	1.5			2.0	2.4			−0.9	2.3		

**(b) Differences between planned and resulting position of the tibia component**													
Sariali	2019	0.1	1.4	−0.4	5.0	0.4	2.7	−0.5	1.2	1.1	4.0	−0.1	2.3
Roh	2013	−0.2	1.4	−2.9	2.4	3.0	2.0	0.0	3.2	0.5	1.8	−2.4	4.5
Crane	2014	0.0	1.6	−5.0	4.0	0.0	2.0	2.0	11.0	−0.2	2.0	−4.0	5.0
		−0.4	1.8	−5.0	3.0	1.1	2.5	−3.0	10.0	−0.3	2.2	−5.0	6.0
De Vloo	2017	0.6	1.7			2.8	2.4			1.4	6.3		

*Note*: Values abstracted and/or adapted to varus +, valgus −/flexion +, extension −/posterior slope +, anterior slope −/internal rotation +, external rotation −.

Fucentese et al. state to reported the accuracy of PSSGs for high tibial osteotomies (HTO) but reported negative mean values [18]. They found a mean difference between plan and postoperative results in the coronal, sagittal, and axial planes of −0.1° (SD 2.3), 1.3° (SD 2.1), and −0.2° (SD 2.3). Jacquet et al. and Chaouche et al. both reported data from a consecutive series of patients treated for medial OA with a HTO between February 2014 and November 2017 in Marseille, France [9, 28]. Jacquet et al. included 71 patients while Chaouche et al. included 100 patients. Both reported the same accuracy of 0.5° (SD 0.6) in the coronal plane and 0.4° (SD 0.8) in the sagittal plane but did not report any data on the accuracy in the axial plane.

Kerens et al. assessed the use of PSSG in UKA and found a mean deviation between preoperative plan and postoperative result of 1.8° in the coronal, 2° in the sagittal and 1° in the axial plane for the femoral component [30]. The postoperative alignment of the tibial component deviated on average 2.5° in the coronal plane, 3° in the sagittal plane, and 5° in the axial plane from the preoperative plan. Van Genechten et al. assessed the use of PSSGs to prepare a personalized bone allograft wedge for opening‐wedge HTO [54]. However, they did not use PSSGs to perform the HTO, their values are nevertheless interesting and were thus included in the study. Absolute mean deviation between planned and achieved alignment after HTO was 1.1° (SD 0.7°) in the coronal plane and 1.2 (SD 1.2°) in the sagittal plane.

### Trauma surgery

One study was selected within the field of trauma surgery [51]. Sun et al. reported on the use of PSSGs in the treatment of complex distal femoral fractures. All variables were significantly superior in the template‐guided group compared to the conventional group. Femoral length difference, anatomical lateral distal femoral angle difference, anatomical posterior distal femoral angle difference, and femoral anteversion angle difference were reported in the same order as follows: 3.31 mm (SD 1.53); 1.57° (SD 0.72); 1.95° (SD 0.78); and 2.52° (SD 1.0).

### Comparison of accuracy between different anatomic regions

**Figure 6:**
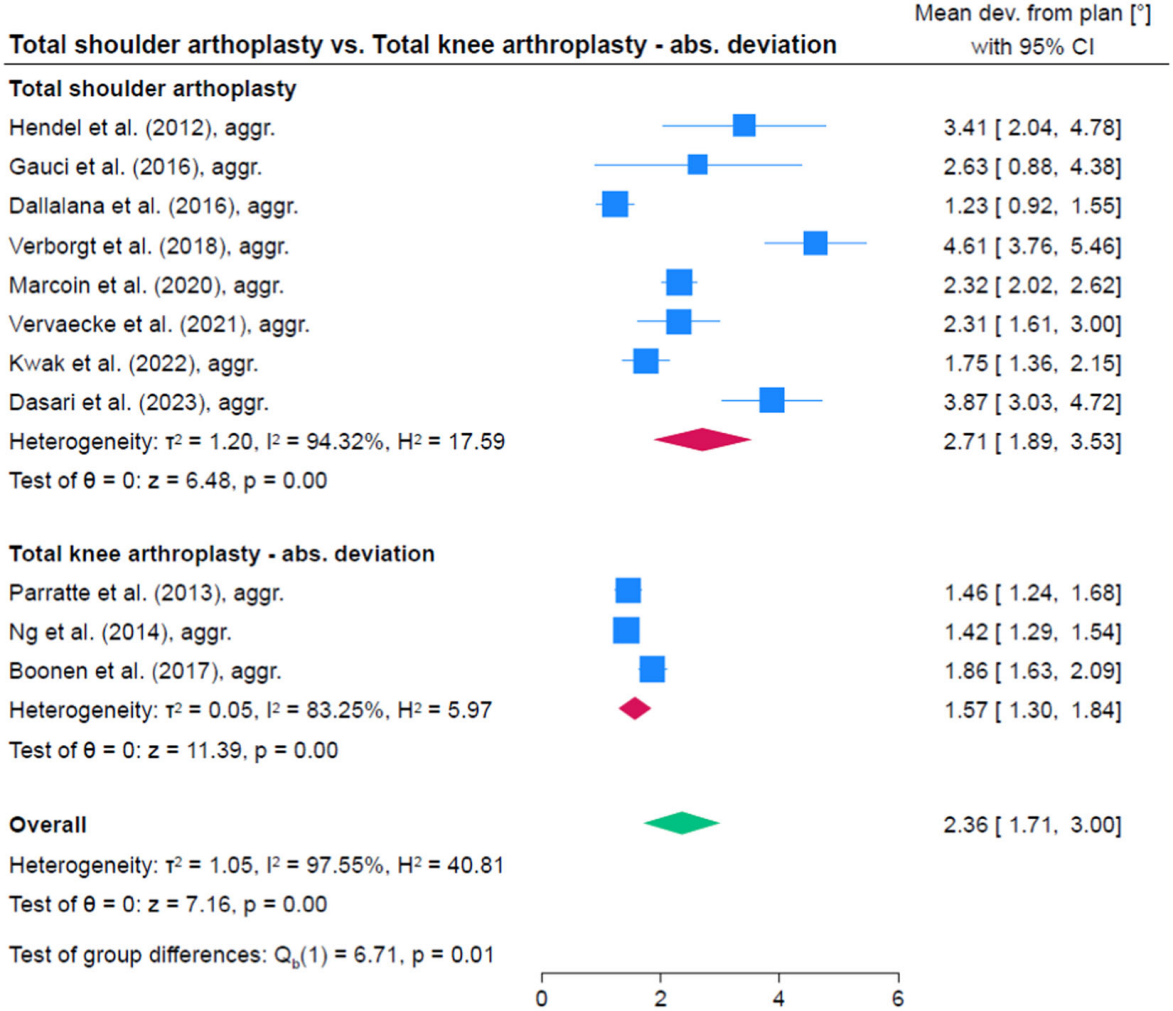
Comparison of accuracy between total shoulder arthroplasty and total knee arthroplasty. CI, confidence interval.

**Figure 7:**
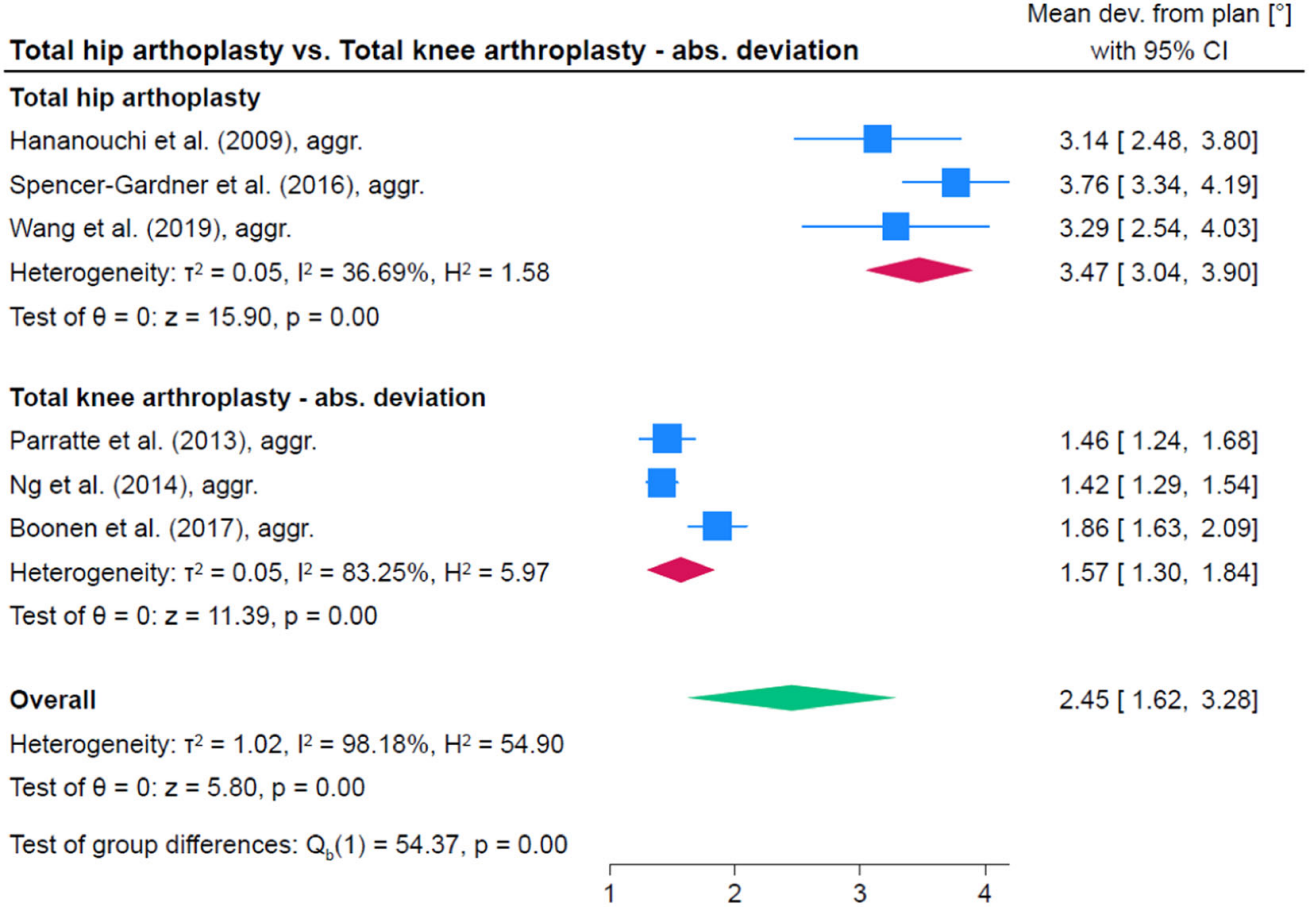
Comparison of accuracy between total knee arthroplasty and total hip arthroplasty. CI, confidence interval.

**Figure 8:**
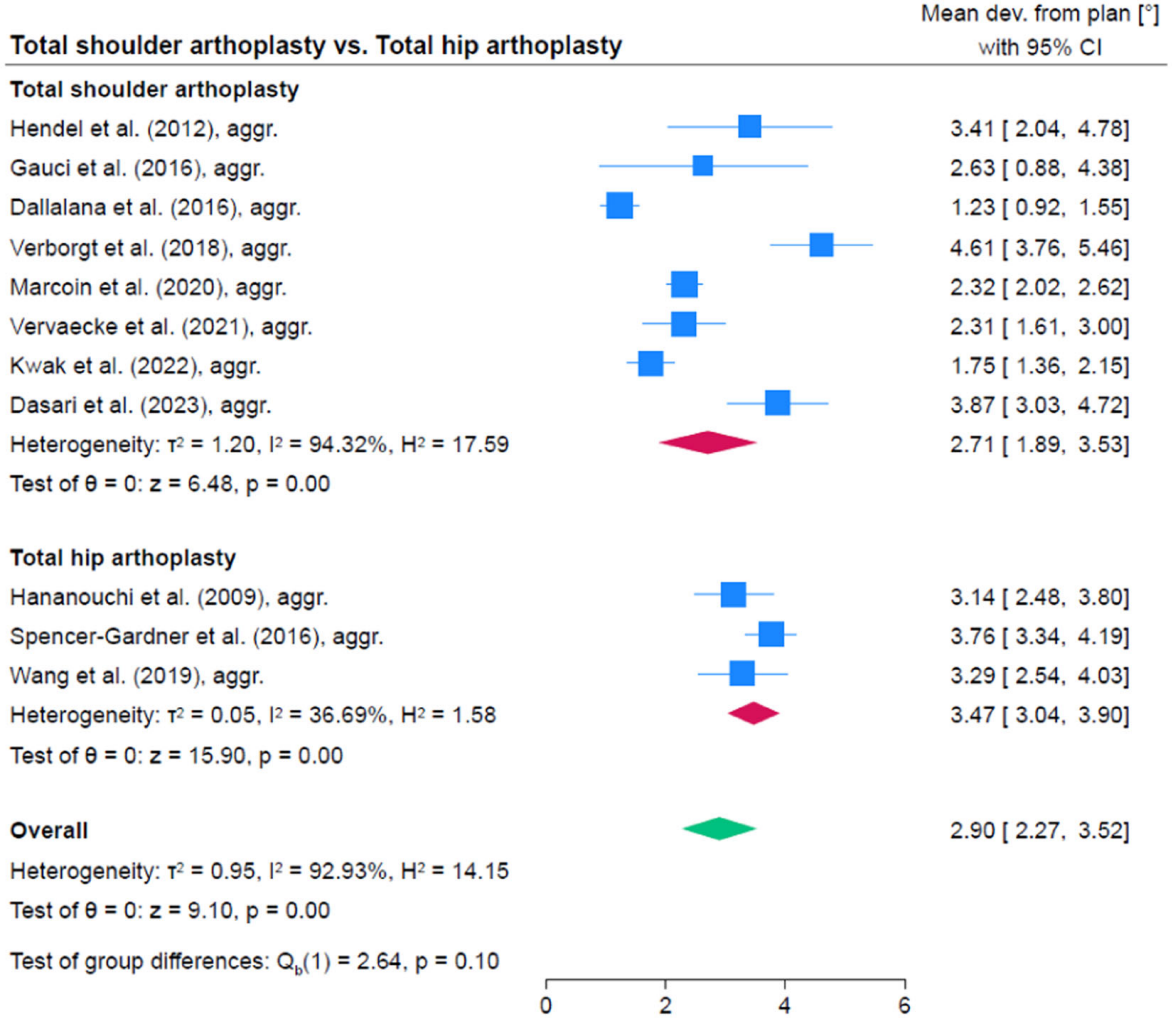
Comparison of accuracy between total shoulder arthroplasty and total hip arthroplasty. CI, confidence interval.

Accuracy was significantly higher in TKA compared to TSA (1.0°, 95% CI: 0.1; 1.9, *p* = 0.035) as well as compared to THA (1.9, 95% CI: 1.4; 2.4, *p* < 0.001). The accuracy was comparable in TSA and THA (0.9, 95% CI: −1.9; 0.0, *p* = 0.056). A comparison between PSSGs provided by PPC and traditional manufacture was not done because there were too few studies per anatomical region and an overall comparison seemed inadequate given the difference in accuracy between anatomic regions. Figures 6, 7, 8 show comparisons between TKA, THA and TSA.

## DISCUSSION

The most important finding of this study is that the accuracy of PSSGs is inadequately evaluated and reported in the literature. The majority of studies evaluating the accuracy of PSSGs are based on conventional radiographs. Measurements based on conventional radiographs are prone to error, and not all relevant parameters can be measured (e.g., tibial component rotation in TKA) [2, 25, 29]. Only a few studies used CT images and they often reported only a part of the relevant data. For example, there are four studies evaluating the accuracy of PSSGs for reaming and cup placement in THA but none evaluated the difference between planned and resulting centre of rotation (rather only inclination/version). Likewise, numerous studies evaluated the accuracy of PSSGs in TKA. However, only nine reported all relevant parameters. Furthermore, four of these nine studies report negative mean differences between preoperative plans and postoperative results [11, 15, 45, 47]. This seems odd since mathematically, a measurement of accuracy cannot be less than 0 (i.e., no deviation). Obviously, the intention behind this denotation was to indicate the direction of deviation, for example, a deviation of the actual position of the femur component to the planned position in a direction of varus in the coronal plane. This is undoubtedly of high clinical relevance but it seems something inherently different from the accuracy of PSSGs in TKA. However, a negative value might be interpreted as a measure of accuracy if the deviation consistently occurs in the same direction across all cases (e.g., if the femur component is consistently in varus compared to the planned position). To account for this possibility, studies reporting negative values were screened again by two experienced orthopaedic researchers (Andreas Hecker and Silvan Hess). Yet based on the available data, this was not the case in any of the above‐mentioned studies. A similar problem was found in spine surgery, where either negative values were reported or only the average planned and postoperative angle were reported. This made it impossible to assess/extract the accuracy of the guides and therefore only four studies could be included, despite a high number of initially identified studies. In conclusion, one of the most frequently cited advantages of PSSGs, improved accuracy compared to conventional instruments, is not as well documented in the literature as expected. More studies investigating the accuracy of PSSGs are needed and they should fulfil the following criteria: (1) analysis should be based on imaging modalities allowing for 3D reconstruction, (2) deviation analysis should be performed by superimposing preoperative plan and postoperative result, (3) deviation analysis should include orientation all planes (e.g., sagittal, coronal and axial) and position (e.g., entry point position) and (4) accuracy should be reported as deviation between planned and resulting position (Mean of absolutes of all differences). Additionally, overall accuracy might be reported as ‘root mean square error’ and the direction of deviation might be indicated by plotting targeted position versus actual position/orientation.

Despite all these limitations and the high heterogeneity of the studies, our meta‐analyses suggest a high accuracy of PSSG in trauma and orthopaedics. Pooled mean deviations between planned and actual position were less than 3.5° in all anatomical regions, which appears to be sufficiently accurate for most orthopaedic procedures.

Another important finding of this study was that the accuracy of PSSGs seems to vary significantly between different anatomical regions. A difference was expected because some anatomical regions seem more amenable to the use of PSSGs than others. However, the difference appears to be greater than expected. The pooled absolute mean difference between preoperative plan and postoperative result in all planes was almost 2° greater in the THA group compared to the TKA group. This difference seems small but should be interpreted with respect to an overall pooled accuracy of 1.82° for TKA. It is therefore important to distinguish between different anatomical regions/surgeries when discussing the accuracy of PSSGs.

The accuracy of PSSGs in TSA has been well evaluated and PSSGs have been found to improve overall accuracy and reduce the rate of outliers compared to the conventional technique [4, 57]. This study further strengthens the value of PSSGs in TSA because all pooled mean values were well below the clinically proposed cut‐off value of 10° for a malpositioned component despite the stricter inclusion criteria compared to previous analyses [23, 27]. The use of PSSGs thereby seems more advantageous in patients with a retroverted and/or damaged glenoid [23, 55]. Yet, if this increased accuracy leads to improved clinical outcomes is unknown since large randomized control trials are missing and small case series reported no or only a small difference in outcome [16, 26].

The use of PSSGs in spine surgery seems limited to pedicle screw placement during spondylodesis and is primarily driven by the desire to reduce iatrogenic damage to neurovascular structures. It thus differs slightly from the primary reason for the use of PSSGs in other regions. Angular deviation and displacement should therefore be interpreted in relation to pedicle breach rates (PBRs). A pooled mean angular deviation of 2.6° and a displacement of 0.84 mm found in this study thereby seems sufficient since only 3.8% of all screws violated the pedicle cortex (all less than 4 mm) and no iatrogenic damage to neurovascular structures was reported. This PBR seems relatively low compared to rates reported for the freehand technique with fluoroscopic assistance (0%−33.1%) and without (13.1%−20%) [37]. There are only two randomized control trials comparing PBRs between a fluoroscopic‐assisted freehand technique and PSI [37, 50]. Matsukawa et al. found a significantly lower rate in the PSI group, while the study by Spirig et al. had to be discontinued after 24 patients because the superiority of the PSI technique was not sufficiently, clinically relevant to justify continuation. The PBR for PSI found in this study seem comparable to other advanced surgical techniques such as robotic‐assisted [32, 46] or CT‐navigated surgery [31, 34]. Last but not least, it should be noted that two of the three studies included in the meta‐analysis evaluated a commercially available product (Medacta MySpine) in the cervical spine, and thus our results might be biased.

The most common application of PSSGs in orthopaedics is in TKA, probably due the controversial topic of alignment. PSSGs are advocated for enhancing alignment accuracy, reducing outlier, and thus leading to an improved clinical outcome and survivorship. Multiple meta‐analyses have explored their effectiveness compared to traditional techniques, but the findings are inconclusive [21, 35, 52]. The pooled mean deviation for all planes from the planned alignment was 1.6°, which is higher than previously reported by meta‐analyses. This difference is most likely due to the rigorous inclusion criteria and exclusion of studies with negative mean values from the meta‐analysis. However, it seems sufficient given the historically promoted safe zone of approximately ±3° from the mechanical axis. Yet, it might not be significantly better than conventional instrumentation [39, 42]. Parratte et al. compared PSI and conventional instrumentation and reported no difference in accuracy. Ng et al. reported an improved accuracy for PSI for the coronal alignment of the tibia and the rotational alignment of both tibia and femur. Accuracy of conventional instrumentation varied between 1.6° (Femur—sagittal) and 16.9° (Tibia—axial) but averaged around 2° in the study by Ng et al., while Parratte did not report any deviations for the conventional group. Again, it should be highlighted that two of the three studies included evaluated the same commercially available product (PSI for NexGen® LPS‐Flex mobile, Zimmer, Warsaw), and our results thus might be biased.

The use of PSSGs in THA is mostly limited to patients with an abnormal anatomy, but may become more common with the growing interest in the effect of spino‐pelvic motion on in vivo functional component alignment. Two of the four included studies evaluated the use of PSSGs in dysplastic hips and reported satisfying results. Interestingly, similar to the results reported for TSA, the benefits of PSSGs were more pronounced in patients with abnormal anatomy (e.g., higher degree of dysplasia). The two remaining studies evaluated the use of a commercially available product (Optimized Positioning System [OPS], Corin Ltd) in which PSSGs are used to transfer patient‐specific kinematic planning to the operating room. As noted above, no data were available regarding deviation between planned and resulting centre of rotation.

Finally, yet importantly, there is a lack of transparency regarding the used printing technologies, printer and resin. Five out of nine studies using PPGSs from PPC unit did not report detailed information and most companies only reported minimal data. Yet, this information seems important when assessing the accuracy of PSSGs and not reporting detailed information does not reflect good clinical practice.

This study has several limitations. The analysis may be influenced by publication bias, as studies reporting low accuracy might be underrepresented in the literature. The diverse anatomical sites and types of surgical guides (even in the same anatomic region) introduce a high level of heterogeneity. Separately meta‐analysis was performed for each region to account for this heterogeneity and reduce the risk of bias. The imaging modality (CT or MRI) used to perform the 3D reconstruction was not considered in the meta‐analysis despite results from the literature indicating a difference in accuracy depending on imaging modality and protocol. Imaging modality was not considered due to the small number of studies in each group and the small and clinically not relevant difference between the modalities expected. The technology of 3D‐printed surgical guides is continuously evolving, potentially affecting accuracy. Some older studies thus may not reflect the performance of newer guides. Last but not least, some studies assessed the accuracy of commercially available products in cooperation with the manufacturer, introducing potential bias in favour of those products.

## CONCLUSION

The accuracy of PSSGs depends on the type of surgery but averages around 2–3°. The use of PSSGs seems advantageous in complex cases. Studies investigating the accuracy of PSSGs should report deviations in all planes.

## AUTHOR CONTRIBUTIONS

All authors contributed to the study conception and design. Material preparation, data collection and analysis were performed by Silvan Hess, Julius Husarek and Martin Müller, respectively. The first draft of the manuscript was written by Silvan Hess and Julius Husarek, and all authors commented on previous versions of the manuscript. All authors read and approved the final manuscript.

## CONFLICT OF INTEREST STATEMENT

The authors declare no conflict of interest.

## ETHICS STATEMENT

No ethical approval was needed for this systematic review and meta‐analysis.

## Supporting information

Appendix 1. Final search strategy.

Appendix 2. Quality Assessment Tool for Quantitative Studies by Effective Public Health Practice Project Canada.

## Data Availability

Data will be made available upon reasonable request.
