# Supplementary material for: Applications and accuracy of 3D‐printed surgical guides in traumatology and orthopaedic surgery: A systematic review and meta‐analysis
Source: J Exp Orthop. 2024 Aug 12;11(3):e12096. doi: 10.1002/jeo2.12096 (PMC11317891; doi:10.1002/jeo2.12096)
Supplement: Supplementary file 1 — Appendix 1. Final search strategy. [file JEO2-11-e12096-s001.docx]

# Appendix I – Final search strategy

## Scopus

( ( TITLE-ABS-KEY ( accur* OR precis* OR reliab* OR valid* OR feasib* OR sufficiently OR efficiency OR efficacy OR safe* OR utilit* ) ) AND ( ( TITLE-ABS-KEY ( "3 dimensional" OR "3-D" OR 3d OR "three-dimensional" W/2 ( print* OR plan* OR "virtual plan*" OR model* OR "surg* model*" OR reconstruct* OR correct* OR prototyp* OR template* ) ) ) OR ( TITLE-ABS-KEY ( "three dimensional" OR "3DP additive manufacturing" OR "additive layer manufacturing" OR "computer aided design" OR "computer aided manufacturing" OR "computer aided machining" OR "computer assisted design" OR "computer assisted manufacturing" OR "computer assisted" OR "computer assisted plan*" OR "computer simulation" OR "preoperative plan*" OR "rapid prototyping" ) ) ) AND ( ( TITLE-ABS-KEY ( "patient specific" OR "patient matched" OR "custom made" OR "custom designed" W/2 ( guid* OR drill* OR cut* OR osteotom* OR surg* OR instrument* OR correct* ) ) ) OR ( TITLE-ABS-KEY ( "surg* guid*" OR "cut* guid*" OR "osteotom* guid*" OR "drill guid*" OR "drill template*" OR "guide wire position*" OR "guide wire placement" OR "intraoperative guid*" OR psi OR "correct* osteotom*" ) ) ) AND ( ( TITLE-ABS-KEY ( bone OR fracture W/2 ( surg* OR osteotom* OR procedures OR therap* OR osteosynthesis OR operation* OR reconstruction* OR reduct* OR fixation* OR correct* OR fixation OR reposition OR deformit* OR stabilization* OR fractur* OR malalignment* OR deformit* OR "orthop?edic surg*" OR traumatolog* OR arthroplast* OR "trauma surg*" ) ) ) OR ( TITLE-ABS-KEY ( fractur* OR osteotom* OR correct* OR osteosynthesis OR fixation OR reposition OR "bone surg*" OR "orthop*edic operation*" OR "orthop*edic procedures" OR "orthop*edic surg*" OR "trauma surg*" OR "open reduction internal fixation" OR "ORIF" OR "high osteotom*" ) ) ) ) AND NOT ( TITLE ( maxillofacial OR craniofacial OR mandibula OR mandible OR maxilla* OR jaw OR midface OR "le fort" OR retrognathia OR prognathism OR "facial reconstruction*" OR synostosis OR "free flap*" OR "dental implant*" OR "tooth implant*" OR dental OR orbita OR skull OR head ) )

## Cochrane

#1 MeSH descriptor: [Reproducibility of Results] explode all trees

#2 (accur* or precis* or reliab* or valid* or feasib* or efficiency or efficacy or safe* or utilit*):ti,ab

#3 MeSH descriptor: [Surgery, Computer-Assisted] explode all trees

#4 MeSH descriptor: [Computer-Aided Design] explode all trees

#5 #3 OR #4

#6 "3 dimensional" OR "3-D" OR "3D" OR "three-dimensional" NEAR/2 (print* or plan* (virtual NEXT plan*) or (surg* NEXT model*) or model* or reconstruct* or correct* or prototyp* or template*):ti,ab

#7 ("three-dimensional" or "3DP additive manufacturing" or "additive layer manufacturing" or "computer aided design" or "computer aided manufacturing" or "computer aided machining" or "computer assisted design" or "computer assisted" or "computer simulation" or (preoperative NEXT plan*) or "rapid prototyping"):ti,ab

#8 ("patient specific" OR "patient matched" OR "custom made" OR "custom designed" NEAR/2 guid* or drill* or cut* or osteotom* or surg* or instrument* or correct*):ti,ab

#9 ((surg* NEXT guid*) or (cut* NEXT guid*) or (osteotom* NEXT guid*) or (drill NEXT guid*) or (drill NEXT template*) or ("guide wire" NEXT position*) or "guide wire placement" or (intraoperative NEXT guid*) or PSI or (correct* NEXT osteotom*)):ti,ab

#10 MeSH descriptor: [Musculoskeletal Diseases] explode all trees and with qualifier(s): [surgery - SU, therapy - TH]

#11 [mh “Orthopedic Procedures”] OR [mh "orthopedics"] OR [mh “traumatology”] OR #10

#12 bone OR fracture NEAR (surg* or osteotom* or procedures or therap* or osteosynthesis or operation* reconstruction* or reduct* or fixation* or correct* or fixation or reposition or deformit* or stabilization* or fractur* or malalignment* or deformit* or (orthop?edic NEXT surg*) or traumatolog* or arthroplast* or (trauma NEXT surg*)):ti,ab

#13 (fractur* or osteotom* or correct* or osteosynthesis or fixation or reposition or bone surg* or (orthop?edic NEXT operation*) or (orthop?edic NEXT procedure*) or (orthop?edic NEXT surg*) or (trauma NEXT surg*) or "open reduction internal fixation" or ORIF or (high NEXT osteotom*)):ti,ab

#14 [mh “Maxillofacial Prosthesis”] OR [mh "Maxillofacial Injuries"] OR [mh “Oral and Maxillofacial Surgeons”] OR [mh "Oral Surgical Procedures"] OR [mh "Dentistry"]

#15 (maxillofacial or craniofacial or mandible or maxilla* or jaw or midface or le fort or retrognathia or prognathism or "facial reconstruction" or synostosis or "free flap" or (dental NEXT implant*) or (tooth NEXT implant*) or dental or orbita or skull or head):ti

#16 #1 OR #2

#17 #5 OR #6 OR #7

#18 #8 OR #9

#19 #10 OR #11 OR #12 OR #13

#20 #16 AND #17 AND #18 AND #19

#21 #14 OR #15

#22 #20 NOT #21

<https://www.cochranelibrary.com/advanced-search/search-manager?search=7074497>

## Ovid MEDLINE

exp "reproducibility of results"/
(accur* or precis* or reliab* or valid* or feasib* or sufficiently or efficiency or efficacy or safe* or utilit*).ti,ab.
Surgery, Computer-Assisted/ or exp Computer-Aided Design/
((3 dimensional or 3-D or 3D or three-dimensional) adj2 (print* or plan* or virtual plan* or model* or surg* model* or reconstruct* or correct* or prototyp* or template*)).ti,ab.
(three-dimensional or 3DP additive manufacturing or additive layer manufacturing or computer aided design or computer aided manufacturing or computer aided machining or computer assisted design or computer assisted manufacturing or computer assisted or computer assisted plan* or computer simultation or preoperative plan* or rapid prototyping).ti,ab.
((patient specific or patient matched or custom made or custom designed) adj2 (guid* or drill* or cut* or osteotom* or surg* or instrument* or correct*)).ti,ab.
(surg* guid* or cut* guid* or osteotom* guid* or drill guid* or drill template* or guide wire position* or guide wire placement or intra?operative guid* or PSI or correct* osteotom*).ti,ab.
exp Orthopedic Procedures/ or exp orthopedics/ or exp traumatology/ or exp *Musculoskeletal Diseases/su, th [Surgery, Therapy]
((bone or fracture) adj2 (surg* or osteotom* or procedures or therap* or osteosynthesis or operation* reconstruction* or reduct* or fixation* or correct* or fixation or reposition or deformit* or stabilization* or fractur* or malalignment* or deformit* or orthop?edic surg* or traumatolog* or arthroplast* or trauma surg*)).ti,ab.
(fractur* or osteotom* or correct* or osteosynthesis or fixation or reposition or bone surg* or orthop?edic operation* or orthop?edic procedures or orthop?edic surg* or trauma surg* or open reduction internal fixation or ORIF or high osteotom*).ti,ab.
exp Maxillofacial Prosthesis/ or exp Maxillofacial Injuries/ or exp "Oral and Maxillofacial Surgeons"/ or exp Oral Surgical Procedures/ or exp Dentistry/
(maxillofacial or craniofacial or mandible or maxilla* or jaw or midface or le fort or retrognathia or prognathism or facial reconstruction* or synostosis or free flap* or dental implant* or tooth implant* or dental or orbita or skull or head).ti.
1 or 2
3 or 4 or 5
6 or 7
8 or 9 or 10
13 and 14 and 15 and 16
11 or 12
17 not 18

<https://ovidsp.ovid.com/ovidweb.cgi?T=JS&NEWS=N&PAGE=main&SHAREDSEARCHID=Fw5xZCrqyiMtDXatJ62rCy9vlPJWVHYnE0pBVwSpAGB6Hc4Fv77j3cSDLnjp8XnG>

## Ovid Embase

exp accuracy/ or exp reliability/ or exp validity/
(accur* or precis* or reliab* or valid* or feasib* or sufficiently or efficiency or efficacy or safe* or utilit*).ti,ab.
exp computer aided design/ or exp computer aided manufacturing/
((3 dimensional or 3-D or 3D or three-dimensional) adj2 (print* or plan* or virtual plan* or model* or surg* model* or reconstruct* or correct* or prototyp* or template*)).ti,ab.
(three-dimensional or 3DP additive manufacturing or additive layer manufacturing or computer aided design or computer aided manufacturing or computer aided machining or computer assisted design or computer assisted manufacturing or computer assisted or computer assisted plan* or computer simultation or preoperative plan* or rapid prototyping).ti,ab.
exp surgical implant template/
((patient specific or patient matched or custom made or custom designed) adj2 (guid* or drill* or cut* or osteotom* or surg* or instrument* or correct*)).ti,ab.
(surg* guid* or cut* guid* or osteotom* guid* or drill guid* or drill template* or guide wire position* or guide wire placement or intraoperative guid* or intra-operative guid* or PSI or correct* osteotom*).ti,ab.
exp orthopedic surgery/ or exp traumatology/ or exp musculoskeletal disease/su, th [Surgery, Therapy]
((bone or fracture) adj2 (surg* or osteotom* or procedures or therap* or osteosynthesis or operation* reconstruction* or reduct* or fixation* or correct* or fixation or reposition or deformit* or stabilization* or fractur* or malalignment* or deformit* or orthop?edic surg* or traumatolog* or arthroplast* or trauma surg*)).ti,ab.
(fractur* or osteotom* or correct* or osteosynthesis or fixation or reposition or bone surg* or orthop?edic operation* or orthop?edic procedures or orthop?edic surg* or trauma surg* or open reduction internal fixation or ORIF or high osteotom*).ti,ab.
exp maxillofacial disorder/ or exp jaw diease/ or exp craniofacial surgery/ or exp dental procedure/ or exp prognathia/ or exp orthognathic surgery/ or exp oral surgery/ or exp tooth/
(maxillofacial or craniofacial or mandible or maxilla* or jaw or midface or le fort or retrognathia or prognathism or facial reconstruction* or synostosis or free flap* or dental implant* or tooth implant* or dental or orbita or skull or head).ti.
1 or 2
3 or 4 or 5
6 or 7 or 8
9 or 10 or 11
14 and 15 and 16 and 17
12 or 13
18 not 19

<https://ovidsp.ovid.com/ovidweb.cgi?T=JS&NEWS=N&PAGE=main&SHAREDSEARCHID=1nx9gmfseJM5DISN3XlHu8QXzV8EWksJq86WTiO5S4ancCclbhUpOX93h7ZmJHvtY>
